# Extracellular Matrix Derived from High Metastatic Human Breast Cancer Triggers Epithelial-Mesenchymal Transition in Epithelial Breast Cancer Cells through αvβ3 Integrin

**DOI:** 10.3390/ijms21082995

**Published:** 2020-04-23

**Authors:** Renata Machado Brandão-Costa, Edward Helal-Neto, Andreza Maia Vieira, Pedro Barcellos-de-Souza, Jose Morgado-Diaz, Christina Barja-Fidalgo

**Affiliations:** 1 Laboratory of Cellular and Molecular Pharmacology, Department of Cell Biology, IBRAG, Rio de Janeiro State University, 20551-030 Rio de Janeiro (RJ), Brazil; renata_machado88@hotmail.com (R.M.B.-C.); edwneto@gmail.com (E.H.-N.); 2 Laboratory of Endothelial Cell and Angiogenesis, IBRAG, Rio de Janeiro State University, 20550-900 Rio de Janeiro (RJ), Brazil; deza.jpa@gmail.com; 3 Cellular and Molecular Oncobiology Program, Instituto Nacional de Câncer, 20231-050 Rio de Janeiro (RJ), Brazil; pedrobsouza@gmail.com (P.B.-d.-S.); jmorgado@inca.gov.br (J.M.-D.)

**Keywords:** breast cancer, extracellular matrix, epithelial-mesenchymal transition, integrin signaling, disintegrin

## Abstract

Alterations in the composition and architecture of the extracellular matrix (ECM) can influence cancer growth and dissemination. During epithelial-mesenchymal transition (EMT), epithelial cells assume a mesenchymal cell phenotype, changing their adhesion profiles from cell-cell contacts to cell-matrix interactions, contributing to metastasis. Breast cancer cells present at different stages of differentiation, producing distinct ECMs in the same tumor mass. However, the contribution of ECM derived from metastatic tumor cells to EMT is unclear. Here, we showed the mechanisms involved in the interaction of MCF-7, a low-metastatic, epithelial breast cancer cell line, with the ECM produced by a high metastatic breast tumor cell, MDA-MB-231 (MDA-ECM). MDA-ECM induced morphological changes in MCF-7 cells, decreased the levels of E-cadherin, up-regulated mesenchymal markers, and augmented cell migration. These changes were accompanied by the activation of integrin-associated signaling, with increased phosphorylation of FAK, ERK, and AKT and activation canonical TGF-β receptor signaling, enhancing phosphorylation of SMAD2 and SMAD4 nuclear translocation in MCF-7 cells. Treatment with Kistrin (Kr), a specific ligand of integrin αvβ3 EMT induced by MDA-ECM, inhibited TGF-β receptor signaling in treated MCF-7 cells. Our results revealed that after interaction with the ECM produced by a high metastatic breast cancer cell, MCF-7 cells lost their characteristic epithelial phenotype undergoing EMT, an effect modulated by integrin signaling in crosstalk with TGF-β receptor signaling pathway. The data evidenced novel potential targets for antimetastatic breast cancer therapies.

## 1. Introduction

Breast cancer is a heterogeneous disease with a variety of clinical and histological forms. The diversity of malignant cell subtypes indicates the need to identify different molecular marks and therapeutic approaches for breast cancer [1,2]. Furthermore, breast cancer remains the second cause of cancer-related death in women, most of them due to metastasis [3,4]. Therefore, it is essential to determine how the metastatic process is regulated in order to identify potential targets for repressing it. 

During metastasis, tumor epithelial cells must acquire a migratory profile assuming a mesenchymal phenotype. The epithelial to mesenchymal transition (EMT) encompasses a well-coordinated series of molecular changes that lead epithelial cells to lose E-cadherin expression, acquire increased vimentin, fibronectin, and N-cadherin expression, and rearrange cortical actin structures, increasing their migratory capacity, characteristics of mesenchymal cells [5,6]. Considered the main inductor of EMT in tumor cells, TGF-β—through the activation of their receptors—regulates two different pathways. The non-canonical pathway is related to the activation of focal adhesion kinase (FAK), ERK/MAPK, and AKT signaling. While the canonical pathway is initiated by the phosphorylation of the cytoplasmic signaling molecules, SMAD2 is followed by recruitment and activation of R-SMAD (SMAD3 associated with SMAD2). This complex is phosphorylated and then associates with SMAD4 through the MH2 domain, triggering its translocation to the nucleus. These two signaling pathways regulate the transcription of different genes that culminate in the characteristic morphological and functional changes of EMT [7,8].

The crosstalk between TGF-β receptors and other receptors, such as integrin, can modulate EMT in different cell types [9]. Integrins are transmembrane adhesive receptors that are able to integrate cells into their microenvironment through their binding to the extracellular matrix (ECM) [10]. Integrin activation modifies actin cytoskeleton dynamics, leading to integrin clustering in focal adhesion complexes and tyrosine phosphorylation of FAK [11]. They can regulate different cell functions, including EMT, through their crosstalk with other membrane receptors [9,10]. In human breast cancer cells, αvβ6 and αvβ1 integrins mediate tenascin-C-induced EMT through a pathway that involves FAK phosphorylation and Src activation, a decrease in E-cadherin expression, and increased cell migration [12,13]. The presence of αvβ3 in MCF-7 cells has been reported, and its expression in breast cancer has been described to be important in response to thyroid hormone, controlling tumor growth [14,15,16]. However, the role of αvβ3 integrin in breast cancer progression is still unclear.

The switch from cell-cell contacts to tumor cell-ECM interactions is typical in EMT [17]. ECM is a dynamic three-dimensional combination of proteins, glycoproteins, and proteoglycans [18] that acts as a scaffold, immobilizing soluble factors and coordinating signals transduced through both growth factors and integrin receptors [19]. Tumor-derived ECM can be remodeled by MMPs released by tumor cells, and the modifications in its composition and architecture may influence cancer growth and dissemination [20]. The specific association of cells with the ECM may provide contextual information that controls differentiation, migration, or survival [19]. Therefore, the biochemical and biophysical properties of the ECM should be considered when examining tumor behavior and therapeutic interventions. Studies using isolated ECM components or tumor fragments have highlighted the role of individual ECM proteins on the breast tumor cell EMT-like phenotype, favoring migration and metastasis [4,21,22,23,24,25]. Soluble tenascin C induces EMT in different breast cancer cell lines through alpha V integrin [13]. Additionally, fibronectin is increased in tumors, promoting tumor growth [26], invasion [27], and limiting tumor cell responsiveness to therapy [28].

Although it is known that certain matrix glycoproteins are up- or down-regulated in tumor matrices during cancer cell transformation [25], evidence of the direct effect of breast cancer metastatic tumor-derived ECM on the phenotypic profile of breast cancer cells with an epithelial phenotype is not available in the literature. 

In the present work, we analyzed the effect of a decellularized, multi-component ECM derived from an aggressive human breast cancer cell line (MDA-MB-231) on a human breast cancer cell line with an epithelial phenotype (MCF-7). Our results showed that MDA-ECM triggered EMT in MCF-7 cells, decreasing cell-cell contacts and E-cadherin expression and increasing the expression of mesenchymal cell markers and migration in MCF-7 cells. These effects were mediated by the crosstalk between integrin αvβ3 and TGF-β pathways.

## 2. Results

### 2.1. Differences in the Composition among Matrices Produced by MCF-7 and MDA-MB-231 Cells 

**Figure 1:**
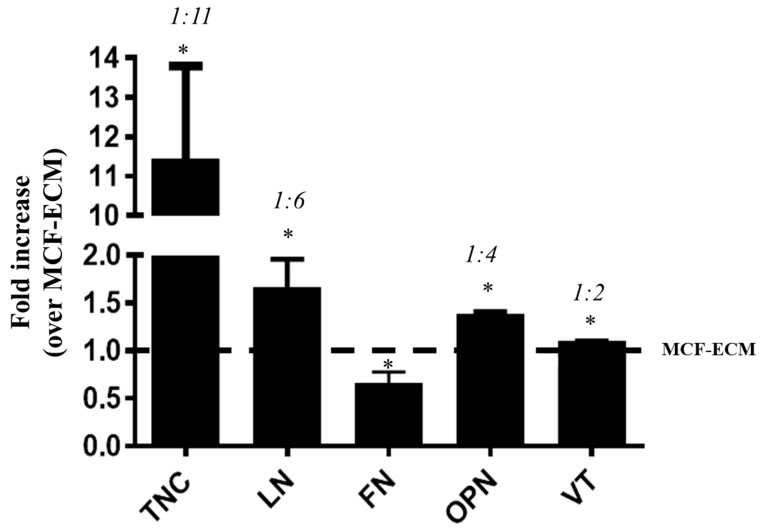
Differences among breast cancer cell-derived matrices. MCF-7 or MDA-MB-231 cells were grown in standard conditions for 72 h, and the respective derived extracellular matrices (ECMs) were obtained, as described in the Methods section. The contents of tenascin-C (TN-C), laminin (LN), fibronectin (FN), osteopontin (OPN), and vitronectin (VT) were assayed by indirect ELISA. The matrix protein content of MDA-ECM is shown as the fold increase relative to MCF-ECM. Numbers at the top of the plots represent an optical density ratio ranging from MCF-ECM:MDA-ECM. The plot is the mean of 3 independent experiments (* *p* < 0.05).

First, we identified matrix glycoproteins in ECMs produced by MCF-7 and MDA-MB-231 cell cultures. Data analysis showed that the two tumor cell-derived ECMs strongly differed in terms of the amounts of some ECM proteins. Relative to the content found in MCF-ECM, the matrix produced by MDA-MB-231 cells presented increased levels of tenascin-C (TN-C) and laminin (LN) and a less effective increase in osteopontin (OPN) and vitronectin (VT). Optical density ratio ranging from MCF-ECM:MDA-ECM was 1:11 fold in TN-C, 1:6 fold in LN, 1:4 fold in OPN, 1:2 fold in VT; and lower amounts of fibronectin (FN) 1:0.6 fold in FN (Figure 1).

### 2.2. Interaction with MDA-ECM Induced EMT-Associated Changes in MCF-7 Cells

**Figure 2:**
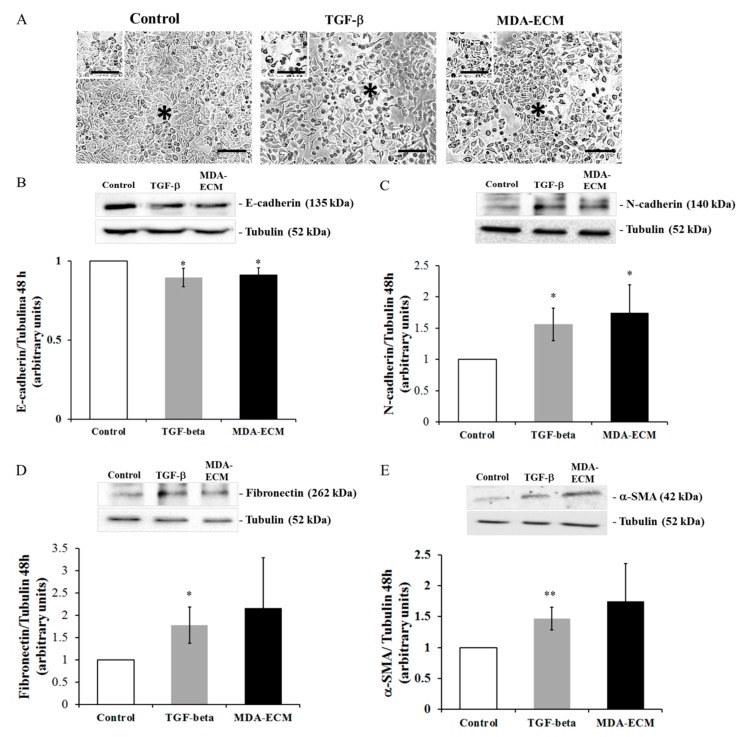
MDA-MB-231-derived ECM promoted a slight decrease in E-cadherin expression in MCF-7 cells in 48 h. MCF-7 and MDA-MB-231 cells were cultured in standard conditions for 48 h, and decellularized ECMs were obtained, as described in Methods. MCF-7 cells were cultured onto MDA-ECM or onto their own matrix with TGF-β1 (10 ng/mL) for 48 h. (**A**) Cell morphology was analyzed, and representative images were obtained at 40× magnification. A black asterisk indicates lost or remaining intercellular connections. Scale bar: 20 µm (**B**–**E**) Lysates of MCF-7 cultured as described for 48 h were immunoblotted with anti-N-cadherin (**B**), anti-α-SMA (**C**), anti-fibronectin (**D**), and anti-E-cadherin (**E**) antibodies. The results are shown as the mean fold increase relative to the control (MCF-ECM), and bars represent the mean ± SD calculated from 3 individual experiments (* *p* < 0.05 and ** *p* < 0.01).

Specific ECM proteins have been reported to induce morphological changes in MCF-7 cells, triggering EMT [12,13,23,29,30,31]. The time of treatment has been reported to be critical for inducing EMT, varying with the stimulus. We observed that, after 48 h, while the MCF-7 cells treated with TGF-β1 changed their morphology, losing cell-cell contacts (*), the cells cultured on their own matrix (MCF-7-ECM), used as controls, were arranged as large clusters, with tight intercellular connections (*). The MCF-7 cells cultured onto MDA-ECM also presented an arrangement in large clusters more similar to controls but different from TGF-β-treated cells since they seemed to maintain looser intercellular connections (*) (Figure 2A). Besides, Figure 2B–E show that MCF-7 cells seeded on MDA-ECM for 48 h presented a slight decrease in E-cadherin and an increase in N-cadherin expression when compared to control (Figure 2B). No differences in the expression of fibronectin or α-SMA were observed. 

**Figure 3:**
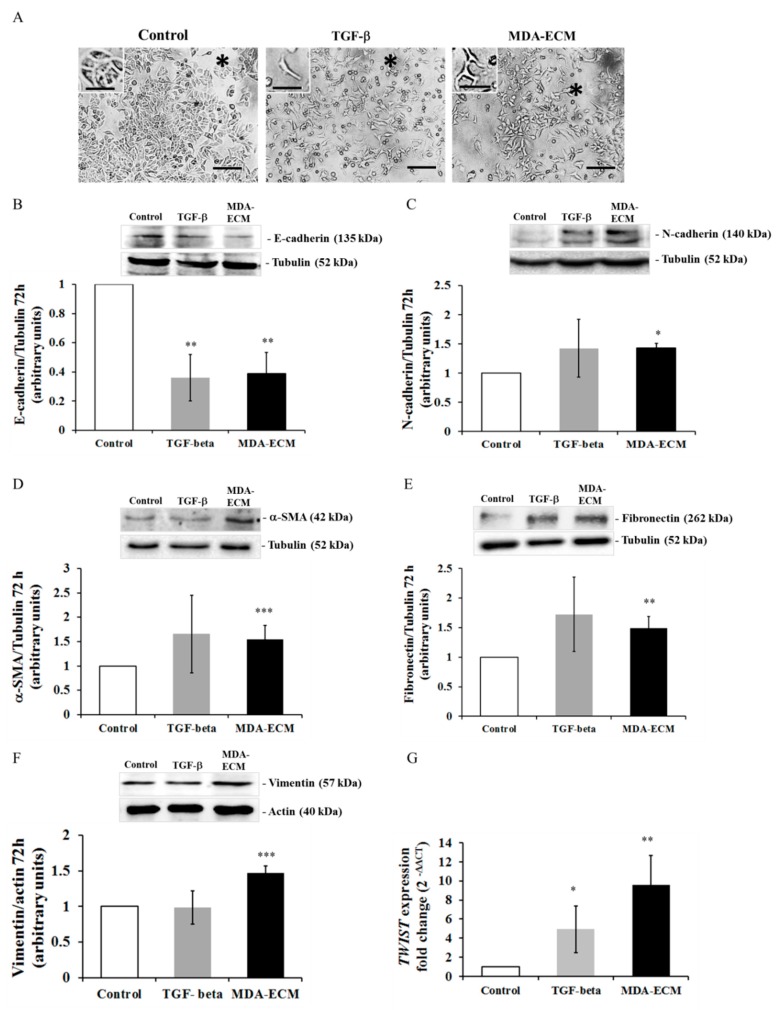
MDA-MB-231-derived ECM triggered morphological and phenotypical changes related to epithelial-mesenchymal transition (EMT) in MCF-7 cells after 72 h. MCF-7 and MDA-MB-231 cells were cultured in standard conditions for 72 h, and decellularized ECMs were obtained, as described in Methods. MCF-7 cells were cultured onto MDA-ECM or onto their own matrix with TGF-β1 (10 ng/mL) for 72 h. (**A**) Cell morphology was analyzed, and representative images were obtained at 40× magnification. A black asterisk indicates lost or remaining intercellular connections. Scale bar: 20 µm (**B**–**F**) Lysates of MCF-7 cultured as described for 72 h were immunoblotted with anti-E-cadherin (**B**), anti-N-cadherin (**C**), anti-α-SMA (**D**), anti-fibronectin (**E**), and anti-vimentin (**F**) antibodies. The results are shown as the mean fold increase relative to the control (MCF-ECM), and bars show the mean ± SD calculated from 3 individual experiments (* *p* < 0.05, ** *p* < 0.01 and *** *p* < 0.001). (**G**) MCF-7 cells were cultured for 48 h on MCF-ECM with or without TGF-β1 or MDA-ECM, and the expression of TWIST mRNA was analyzed. β-Actin was used as a housekeeping gene. The results are shown as the mean ± SD calculated from 3 individual experiments (* *p* < 0.05).

However, MCF-7 cells seeded on MDA-ECM for 72 h presented an increase in the number of cells with a spindle-shaped morphology compared with that observed after 48 h (Figure 3A). Besides, MCF-7 cells cultured onto MDA-ECM for 72 h showed increased expression of N-cadherin, α-SMA, fibronectin, and vimentin when compared to the control group (Figure 3C–F). Notably, after 72 h, MDA-ECM, and also positive control, induced, in a more prominent manner, a decrease in E-cadherin expression (Figure 3B), accompanied by an increase in the expression of the transcriptional repressor *TWIST* (Figure 3G). For these reasons, we decided to use the time of 72 h in this study. 

**Figure 4:**
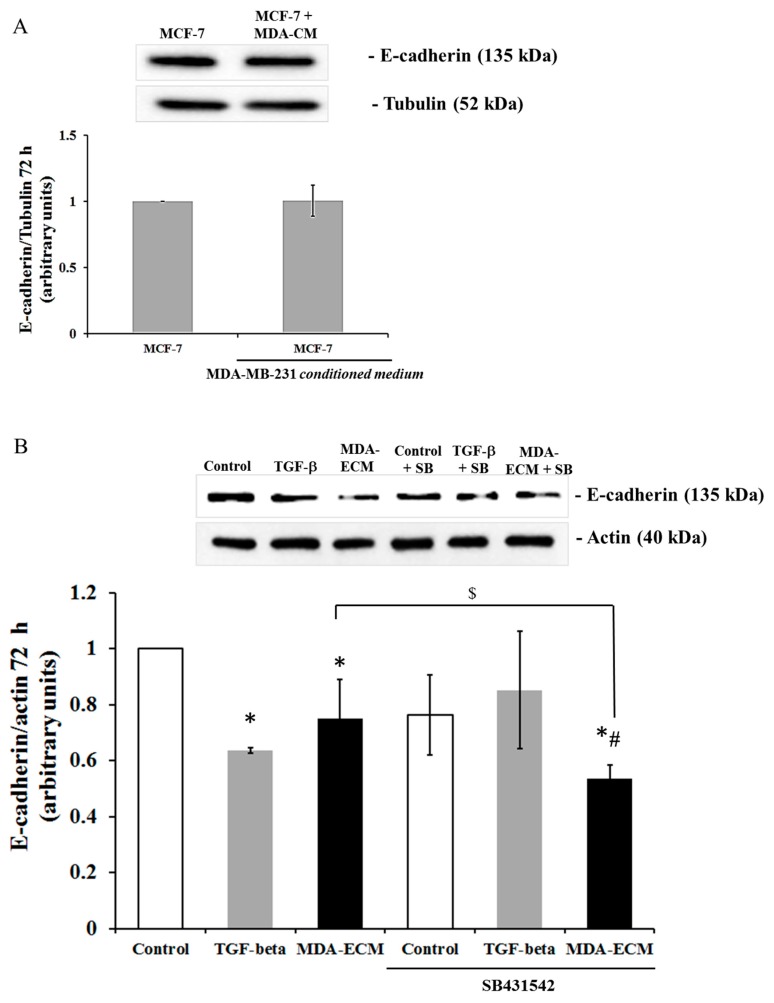
MDA-MB-231-derived ECM decreased E-cadherin expression in MCF-7 cells independent of TGF-beta receptor activation. MCF-7 and MDA-MB-231 cells were cultured in standard conditions for 72 h, and decellularized ECMs were obtained, as described in the Methods. (**A**) MCF-7 were seeded on their own matrix for 72 h with or without conditioned medium from MDA-MB-231 cultures. Cell lysates were immunoblotted with anti-E-cadherin and anti-tubulin antibodies. (**B**) MCF-7 cells were seeded on decellularized MCF-ECM with or without TGF-β1 or MDA-ECM in the presence or absence of the TGF-β receptor inhibitor SB431542 (15 µg/mL) for 72 h. Cell lysates were immunoblotted with anti-E-cadherin and anti-actin antibodies. The results are shown as the mean fold increase relative to the MCF-ECM (* *p* < 0.05); MCF-ECM treated with SB431542 (# *p* < 0.05) and MDA-ECM without inhibitor ($ *p* < 0.05) were calculated from 3 individual experiments.

ECM can anchor a variety of molecules, including TGF-β [17], which can induce EMT changes in certain breast cancer cells [13]. Thus, we considered whether the TGF-β produced by MDA-MB-231 cells could be, in part, present on MDA-ECM, regulating EMT changes observed in MCF-7 cells. MDA-MB-231 cells secreted more amounts of TGF-β in the conditioned medium than MCF-7 cells [32,33]. To investigate the possibility that TGF-β secreted by MDA-MB-231 cells change MCF-7 phenotype, cells were cultured for 72 h with the conditioned medium harvested from MDA-MB-231 cell cultures. We did not observe any decrease of E-cadherin expression in MCF-7 cells treated with the MDA-MB conditioned medium (Figure 4A). Furthermore, treatment with 15 µg/mL of an inhibitor of the TGF-β type I receptor (SB431542) did not interfere with the effect of MDA-ECM on E-cadherin expression in MCF-7 cells (Figure 4B). Consistently, SB431542 inhibited the direct effect of TGF-β1 on E-cadherin levels in MCF-7 cells (Figure 4B). The efficiency of the TGF-β inhibitor has been tested (Figure S1A,B). These sets of data suggested that the MCF-7 cell-matrix contact with MDA-ECM could be contributing to the decrease in E-cadherin expression, favoring EMT in MCF-7 cells.

### 2.3. Interaction with MDA-ECM Increased MCF-7 Cells Migratory Capacity

**Figure 5:**
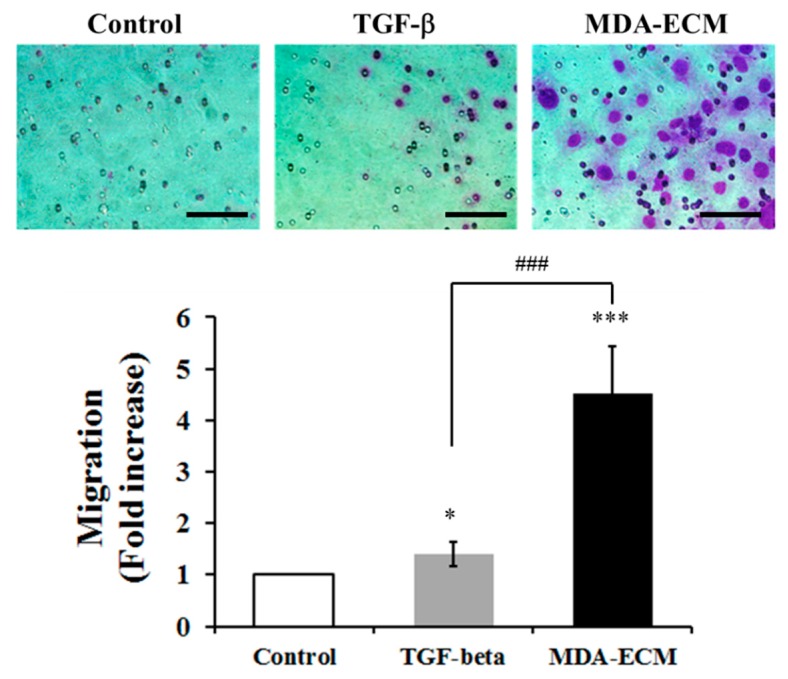
Effect of MDA-ECM on the migratory capacity of MCF-7 cells. MCF-7 and MDA-MB-231 cells were cultured in standard conditions for 72 h, and decellularized ECMs were obtained, as described in the Methods. MCF-7 cells were cultured for 72 h on decellularized ECM derived from MDA-MB-231 or MCF-7 cells with or without TGF-β1. After that, cells were trypsinized, seeded on Transwell chambers, and allowed to migrate toward FBS-enriched medium (10%) for 72 h. The number of migrated cells was counted, and the results are shown as the fold increase relative to the control MCF-ECM (* *p* < 0.05 and *** *p* < 0.001) and the positive control TGF-beta (### *p* < 0.001) and presented as the mean ± SD calculated from 3 individual experiments. Scale bar: 20 µm.

Loss of cell-cell adhesion structures and polarity leads to increased motility and invasiveness of tumor epithelial cells [6,12,34]. MCF-7 cells are known to have a low capacity of migration in vitro [35]. Therefore, to evaluate the effect of MDA-ECM on MCF-7 migration, we incubated these cells onto their own matrix in the presence or absence of TGF-β or onto MDA-ECM for 72 h, and then these cells were added in transwell inserts. Using the FBS-enriched medium as a chemoattractant, MCF-7 cells were allowed to migrate for 72 h. Figure 5 demonstrates that the previous treatment with TGF-β or with MDA-ECM increased the migration of MCF-7 cells. However, MCF-7 cells that were previously seeded onto MDA-ECM had a most effective increase (4.2 fold increase), when compared with the positive control (1.4 fold increase). 

### 2.4. Interaction with MDA-ECM Triggered Integrin and TGF-β Signaling Pathways in MCF-7 Cells

Alterations in ECM composition can influence the phenotype of different cell types present in the tumor stroma [18]. Integrin coordinates the interactions between cells and ECM, triggering adhesive signaling characterized by focal adhesion kinase (FAK) activation and the recruitment of the actin cytoskeleton; this is followed by a cascade of intracellular signals that are also involved in cell migration, proliferation, and survival, including PI3K/Akt and MAPK/ERK pathway activation [36,37].

**Figure 6:**
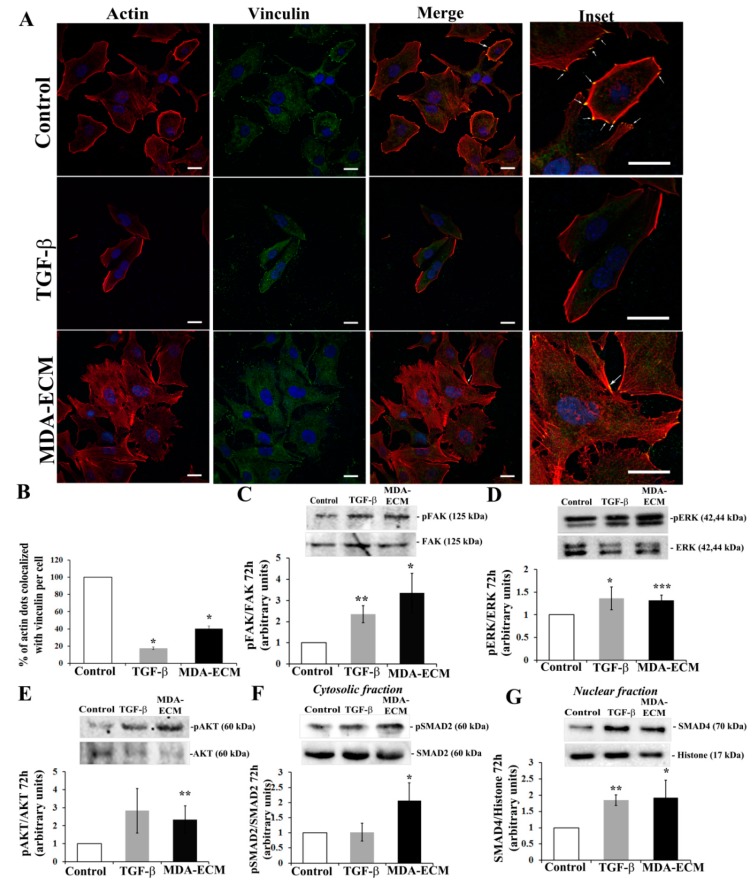
Interaction with MDA-ECM triggered integrin and TGF-β-associated signaling pathways in MCF-7 cells. (**A**) MCF-7 and MDA-MB-231 cells were cultured in standard conditions on coverslips for 72 h, and decellularized ECMs were obtained, as described in Methods. For double staining of F-actin and vinculin (indicated by the white asterisks), cells were marked with rhodamine-conjugated phalloidin (red) and anti-vinculin antibody (green), and nuclei were stained with DAPI. Representative images were captured at 60× magnification. Focal adhesion assembly in control is highly visible in the section at high magnification (insets-right column). Scale bar: 20 µm. (**B**) Representative graph showing the percentage of colocalization of vinculin and actin in comparison to the control MCF-ECM. Cell lysates were immunoblotted with (**C**) anti-pFAK^Tyr397^ and anti-FAK, (**D**) anti-pERK1/2 and anti-ERK1/2, (**E**) anti-pAKT^Ser473^ and anti-AKT, (**F**) anti-pSMAD2 and anti-SMAD2, and (**G**) anti-SMAD 4 and anti-histone (H3) antibodies. The results are shown as the fold increase compared to the MCF-ECM group, calculated from 3 individual experiments (* *p* < 0.05; ** *p* < 0.01; *** *p* < 0.001).

We assessed through confocal microscopy the focal adhesion assembly in MCF-7 cultured onto the tumors-derived matrices, using double staining for F-actin and vinculin that appeared as yellow spots on cell borders (Figure 6A; white asterisks). The interaction with MDA-ECM, as well as the treatment with TGF-β1, impaired focal adhesion formation in MCF-7 cells (Figure 6A,B). However, MCF-7 cells seeded onto MDA-ECM showed major alterations in the cytoskeleton dynamics, presenting stress fibers with lamellipodia and filopodia formation, very characteristics of a migratory profile (Figure 6A, lower panel). This phenotype contrasted with that of MCF-7 cells cultured on MCF-ECM, with or without TGF-β1 that presented a more cortical distribution of F-actin (Figure 6A, upper and middle panels). In parallel, we also showed that MCF-7 cells seeded on MDA-ECM increased FAK^Y397^ phosphorylation (Figure 6C), phospho-ERK1/2 (Figure 6D), and phospho-AKT (S473) (Figure 6E), characterizing the involvement of integrin-associated signaling.

Canonical and non-canonical TGF-β receptor signaling pathways are reported to regulate EMT. The activation of FAK and of PI3K, and AKT pathways, can be also involved in non-canonical activation of the TGF-β receptor [38]. The onset of the canonical TGF-β receptor signaling comprises the phosphorylation of SMAD2 that, together with SMAD 3, triggers SMAD4 translocation to the nucleus [9]. When MCF-7 cells were seeded onto MDA-ECM, we observed, in parallel to integrin signaling activation, an increase in SMAD2 phosphorylation (S465–467) expression (Figure 6F) and increased SMAD4 expression in the cell nucleus (Figure 6G). 

### 2.5. Involvement of αvβ3 Integrin in the EMT of MCF-7 Cells Cultured on MDA-ECM

**Figure 7:**
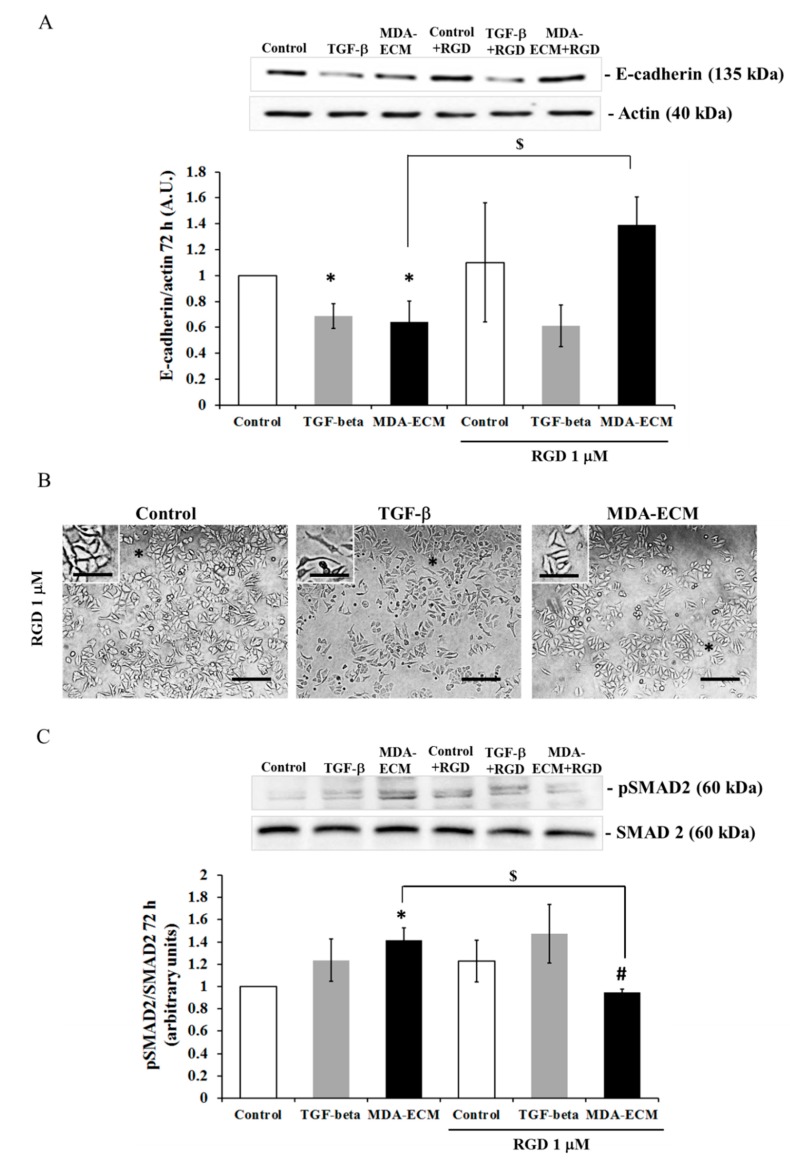
Integrin signaling modulated the effects of MDA-ECM on E-cadherin-mediated adhesion of MCF-7 cells. MCF-ECM and MDA-ECM were obtained, as described in the Methods section. MCF-7 cells were cultured on their own ECM with or without TGF-β1 or on MDA-ECM and treated with Arg-Gly-Asp (RGD) for 72 h (1 µM). Cell lysates were immunoblotted with (**A**) anti-E-cadherin and (**C**) anti-*p*-SMAD2 antibodies. (**B**) Representative images of MCF-7 cell cultures of each experimental group, treated or not with RGD (1 µM), at 40× magnification. A black asterisk indicates lost or remaining intercellular connections. Scale bar: 20 µm. The results are shown as the mean fold increase relative to controls: MCF-ECM (* *p* < 0.05), MCF-ECM + RGD (# *p* < 0.05), and MDA-ECM ($ *p* < 0.05), calculated from 3 individual experiments.

Integrins are reported to have a critical role during the process of EMT through crosstalk with TGF-β receptors [4,8,12,19,39]. To evaluate the role of integrins in the activation of TGF-βR signaling activation, the experiments were performed in the presence of a synthetic tripeptide RGD (Arg-Gly-Asp) that blocks αv integrin activation [40]. Figure 7 shows that the RGD peptide impaired the decrease in E-cadherin levels (Figure 7A) of cells cultured onto MDA-ECM. Supporting the data, the immunofluorescence analysis showed the presence of E-cadherin in the intercellular junctions in MCF-7 cells cultured on MDA-ECM treated with RGD, contrasting with cells not treated with the peptide or stimulated with TGF-β1 (Figure S2A,B). In addition, RGD treatment inhibited SMAD2 phosphorylation only in MCF-7 cells cultured on MDA-ECM and not in MCF-ECM groups (Figure 7C).

**Figure 8:**
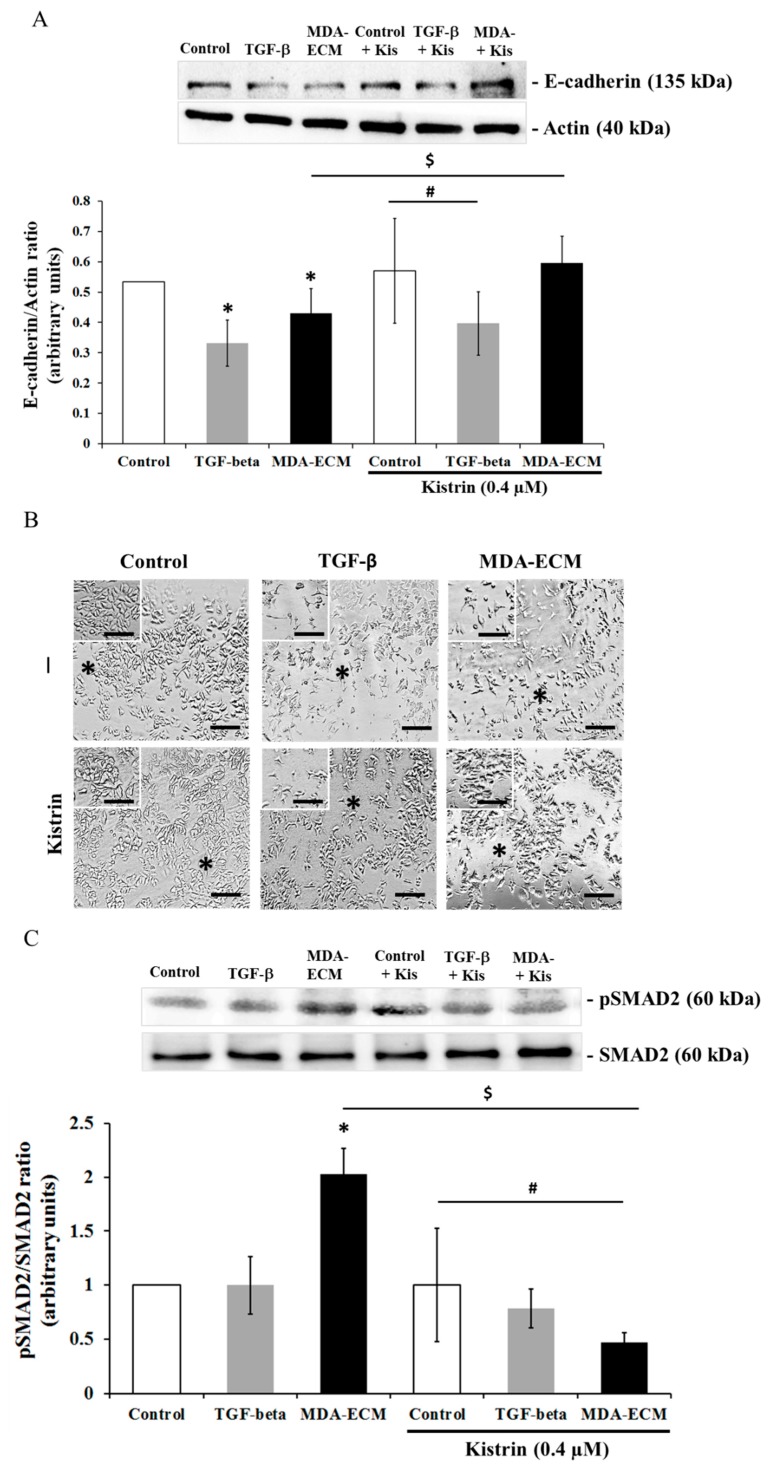
Involvement of integrin αvβ3 in EMT of MCF-7 cells cultured on MDA-ECM. MCF-ECM and MDA-ECM were obtained, as described in the Methods section. MCF-7 cells were cultured on their own ECM with or without TGF-β1 or on MDA-ECM and treated with Kistrin for 72 h (0.4 µM). Cell lysates were immunoblotted with (**A**) anti-E-cadherin and (**C**) anti-*p*-SMAD2 antibodies. The results are shown as the mean fold increase relative to controls: MCF-ECM (* *p* < 0.05), MCF-ECM + Kistrin (# *p* < 0.05),and MDA-ECM ($ *p* < 0.05), calculated from 3 individual experiments. (**B**) Representative images of MCF-7 cell cultures of each experimental group, treated or not with Kistrin (0.4 µM), at 10× magnification. A black asterisk indicates lost or remaining intercellular connections. Scale bar: 20 µm.

The involvement of αv subfamily of integrins has been often associated with EMT in human breast cancer cells [12,41]. To evaluate the involvement of αv integrins as mediators of EMT in our experimental model, MCF-7 cells cultured on their own matrix, with or without TGF-β1 and MDA-ECM, were treated with Kistrin, an RGD-disintegrin that selectively binds to and blocks many functions of αvβ3 integrin [40,42]. The treatment with Kistrin impaired the effect of MDA-ECM on MCF-7 cells, maintaining high levels of E-cadherin expression (Figure 8A) and supporting cell-cell contacts with tight intercellular connections, indicated by the black asterisk (Figure 8B). In contrast, the effect of TGF-β1 in decreasing E-cadherin expression and changing MCF-7 cells’ morphology was not altered in cells treated with Kistrin (Figure 8A,B). Importantly, blocking αvβ3 integrin with Kistrin significantly decreased SMAD2 phosphorylation only in MCF-7 cells cultured on MDA-ECM, but not in control cells treated or not with TGF- β1 (Figure 8C). These data indicated that MDA-ECM-induced EMT-related changes in MCF-7 cells might be driven by αvβ3 integrin-mediated interactions.

## 3. Discussion

In the tumor microenvironment, ECM produced by different tumor cell types varies along cancer progression stages [43]. Evidence has shown that ECM proteins modulate the behavior of cells in the tumor niche, including the ECM produced by tumor cells themselves, providing critical signals that can contribute to cancer development [25]. Despite the complex structure of tumor-produced ECMs, most studies on the role of matrices in cancer have been performed using single ECM proteins or artificial matrices [13,44,45]. Here, we used a natural 3D matrix produced by highly metastatic breast cancer cells (MDA-MB-231) to investigate how it would affect the phenotype of non-metastatic MCF-7 cells, focusing on its capacity to induce EMT.

The transition from an epithelial to a mesenchymal phenotype is a key mechanism in cancer progression whereby tumor cells down-regulate epithelial markers and increase mesenchymal markers, becoming more migratory and acquiring a more aggressive behavior. These changes usually correlate to a higher invasive potential, increased metastasis, and poor patient outcome [46]. Different studies have demonstrated that isolated matrix proteins can influence EMT in some tumors, particularly in breast cancer [9,13,19,23,24,27,39,47,48,49].

Intratumor heterogeneity consists of a major challenge for designing effective therapies, and breast cancers are usually highly heterogeneous, including tumor cells at different stages of differentiation [1,50]. For this reason, one expects that the composition of ECM produced by each cell type may differ and influence the phenotype of neighboring cells present in the tumor mass. We showed that the interaction with the extracellular matrix produced by high metastatic breast cancer cells, MDA-MB-231, is able to induce EMT in MCF-7 cells, an epithelial-type breast cancer cell, increasing mesenchymal marker expression, modifying cell phenotype, and stimulating cell migration. These changes were detected when MCF-7 cells were cultured on MDA-ECM, which, in a comparative analysis, showed a distinct matrix protein composition. The ECM produced by MDA-MB-231 cells presented higher amounts of tenascin-C and laminin and a less effective increase in osteopontin and vitronectin and lower amounts of fibronectin when compared to MCF-ECM. 

The tenascin-C expression is normally sparse in healthy human cells but is rapidly induced in many tissues in response to pathological stress [51]. Up-regulation of tenascin-C correlates with situations of tissue repair, and increased tenascin-C protein levels have been detected in human cancers and are associated with a poor prognosis, higher aggressiveness, and malignancy [52,53]. Soluble tenascin-C has been shown to enhance migration and loss of intercellular adhesion in breast cancer cells [13]. Laminin has been described as a potent adhesive and migratory substrate for metastatic breast tumor cells in vitro, and its higher expression correlates with tumor grade and metastatic potential in vivo [4]. Laminin has also been shown to increase the migration of MCF-7 and MDA-MB-231 cells through a mechanism involving integrin modulation [19]. Osteopontin, whose expression was also augmented in MDA-ECM, has been associated with a poor prognosis in patients with breast cancer [20,54] and with higher metastatic potential in rodent models [55]. Finally, vitronectin, which, along with osteopontin, is a potent ligand of αvβ3 integrin, seems to modulate cancer cell adhesion, migration, and angiogenesis [56]. The presence of these proteins on MDA-ECM is consistent with the increased metastatic potential of MDA-MB-231 cells. Differences in MDA-ECM composition might be responsible for inducing EMT in MCF-7 cells, which assumed morphologic, molecular, and functional profiles typical of a mesenchymal phenotype. After the interaction of those epithelial breast cancer cells with MDA-ECM for 72 h, we observed a decrease in E-cadherin expression and an increase in N-cadherin and other mesenchymal markers, such as α-SMA, vimentin, fibronectin, and *TWIST*. These molecular changes were accompanied by loss of cell-cell contacts and increasing migratory capacity of MCF-7 cells. Noteworthy, after 72 h incubation of MCF-7 cells with TGF-β (used as positive control), compared with MDA-ECM, we observed a less evident effect on EMT markers, which could be explained because TGF-β effects peaked in earlier times and might decrease afterward (Figure 2 and Figure 3).

The extracellular matrix can regulate the availability and activity of many carcinogenic mediators, including TGF-β, through controlled sequestration, presentation, and release [19]. Since the MDA-MB-231 cells could be releasing TGF-β in culture medium [29,30], we argued whether this secreted TGF-β might change the MCF-7 phenotype. We demonstrated that TGF-β, if present in the conditioned medium of MDA-MB-231 cell cultures, was not critical for the induction of EMT in our model. Furthermore, blocking the TGF-β receptor in MCF-7 cells did not modify the EMT induced by MDA-ECM, but impaired the TGF-β effects on EMT protein markers and on changes of epithelial morphology (Figure 4 and Figure S1A,B). Although we could not completely discard the presence of TGF-β and other soluble factors anchored to MDA-ECM, our data strongly evidenced that the direct contact with MDA-ECM could prime MCF-7 cells triggering an EMT more efficiently than in the presence of TGF-β alone.

It has been reported that integrin-mediated cell-matrix interactions can modulate tumor progression, including EMT [4,12,19,39,57]. We showed that MCF-7 cells cultured on MDA-ECM assumed a more activated and migratory profile, with profound changes in cytoskeleton dynamics. These alterations in cell morphology were accompanied by the activation of integrin signaling pathways, with an increase in the phosphorylation of FAK, AKT, and ERK in MCF-7 cells cultured on MDA-ECM.

Activated FAK acts as a scaffold protein with binding sites for several classes of signaling molecules, including PI3K/AKT and MAPK/ERK, that regulate cell migration and survival and are involved in the non-canonical TGF-β signaling pathway [8,58,59]. In addition, recent studies have shown that AKT and ERK are involved in EMT, participating in the crosstalk between the integrin and TGF-β receptor pathways [8,9,60,61,62]. Increased amounts of type I collagen correlate with integrins, switching to a high-affinity ligand-binding state, increasing FAK- and ILK-signaling activity, and promoting EMT in tumor pancreatic cells, for example [63]. Our data showed that MDA-ECM induced an increase in integrin signaling activation parallel with an increase in SMAD2 phosphorylation and subsequent translocation of SMAD4 to the nucleus in MCF-7 cells, suggesting crosstalk between integrin and TGF-β receptor signaling pathways. These effects of MDA-ECM were more evident in MCF-7 cells after 72 h in contact with the matrix, occurring later than the direct effects of TGF-β, which were more prominent at early times (Figure 2). This is rather interesting that EMT could be further improved in epithelial tumor cells after their contact with the ECM produced by a higher metastatic cell present in the same tumor mass. 

To investigate the role of integrins in the effects induced by MDA-ECM, the cells were treated with RGD peptide, an unspecific ligand of integrins, or with Kistrin, a selective ligand of αvβ3 integrin [51,52], both able to bind and block many integrin functions [40]. The treatment of MCF-7 cells seeded on MDA-ECM with Kistrin or RGD preserved cell-matrix and cell-cell contacts, but impaired the decrease of E-cadherin expression and SMAD2 phosphorylation, revealing transactivation between integrins and TGF-β receptor to drive EMT. Interestingly, it is already known that integrin αvβ3 is expressed in epithelial cells in cancer tissues and acts as a driver of cancer progression, stemness, and metastasis. So, this integrin possibly, in conjunction with growth factor receptors, can regulate proliferation and migration of cancer cells influenced by tenascin-C protein [64,65,66]. Noteworthy, tenascin-C is the most expressed extracellular matrix in MDA-ECM. Therefore, tenascin-C expression accompanied with vitronectin and laminin in MDA-ECM, which can bind to αvβ3, could have a crucial role for triggering EMT in MCF-7 cells.

In line with these observations, some studies have correlated the different expression of extracellular matrix proteins and its stiffness to aberrant activation of the TGF-β pathway, accompanied by an expression of determined integrins, with enhancing cancer cell stemness and invasion [67,68,69]. In addition, tumor-initiating cells are more resistant to detachment-induced anoikis and exhibit phenotypic plasticity to move between epithelial to mesenchymal states [69,70]. Tumor-initiating cells are the driver for drug resistance, cancer relapses, and metastasis, which remains to occur in some cases of breast cancer [69,70,71]. Taken together, this evidence suggests a tight association among the EMT phenotype induced by MDA-MB-231-derived extracellular matrix, through αvβ3 integrin activity and tumor-initiating cells, but further investigation is required in future studies.

## 4. Material and Methods

### 4.1. Reagents

Culture reagents—bovine serum albumin (BSA), penicillin, streptomycin, trypsin, Dulbecco’s Modified Eagle Medium (DMEM); Arg-Gly-Asp (RGD); protease inhibitors, phenylmethylsulfonyl fluoride (PMSF), leupeptin, and ribonuclease A (RNase A); trypan blue and specific inhibitor of transforming growth factor-beta superfamily type, SB431542 (Sigma-Aldrich, St. Louis, MO, USA, #S4317), was diluted in DMSO to obtain a stock concentration of 1000 µM/mL. Recombinant human TGF-beta was from Peprotech (Ribeirão Preto, SP, Brazil). Fetal bovine serum (FBS) was purchased from Cultilab (Campinas, SP, Brazil). BCA protein assay kit was purchased from Thermo Scientific. TRITC-labeled phalloidin was obtained from Sigma-Aldrich (St. Louis, MO, USA), DAPI ProLong^®^ was obtained from Invitrogen (Carlsbad, CA, USA), and Alexa Fluor 555 and 488 were obtained from Invitrogen, Paisley, UK. PVDF membranes and Rainbow^TM^ molecular weight markers were from GE Healthcare (San Francisco, CA, USA), and the ECL system (SuperSignal West Pico chemiluminescent substrate kit) was from Pierce Biotechnology (Rockford, IL, USA). High capacity cDNA reverse transcription kit was purchased from Applied Biosystems. The RNeasy mini kit and SYBR-green were obtained from Qiagen. Primers for *TWIST* (Forward 5′-GGCACCATCCTCACACCTCT-3′; reverse 3′-TGGCTGATTGGCACGACCT-5′) and *β-actin* (Forward 5′-TACAATGAGCTGCGTGTGG-3′; reverse 3′-TAGCACAGCCTGGATAGCAA-5′) were purchased from IDT technologies. The panoptic staining kit was from LB Laborclin (Pinhais, PR, Brazil). Kistrin (Kr; Lot 44H4073), isolated from *Agkistrondon rhodostome* venom, was purchased from Sigma Chemical Co. (St. Louis, MO, USA).

### 4.2. Antibodies 

Primary antibodies: rabbit polyclonal anti-p-FAK^Tyr397^ and anti-tenascin-C (Invitrogen, Carlsbad, CA, USA); mouse monoclonal anti-osteopontin, anti-α-SMA, goat monoclonal anti-Akt1, and rabbit polyclonal anti-vitronectin (Santa Cruz Biotech., Santa Cruz, CA, USA); rabbit polyclonal anti-fibronectin (Dako, Denmark, SC, USA); rabbit monoclonal anti-FAK (Abcam, Cambridge, MA, USA); rabbit polyclonal anti-laminin, mouse monoclonal anti-α-tubulin, and anti-vinculin (Sigma-Aldrich Co., St. Louis, MO, USA); rabbit monoclonal anti-E-cadherin, anti-SMAD2, anti-p-ERK1/2^Thr202/204^, anti-p-SMAD2^Ser465/467^, and polyclonal anti-actin, anti-ERK 1/2, anti-SMAD4, and anti-N-cadherin (Cell Signaling Technol., Danvers, MA, USA); mouse monoclonal anti-p-Akt^Ser473^, anti-actin, and rabbit monoclonal anti-histone (H3) (Millipore, Billerica, MA, USA). Secondary biotin-conjugated antibodies against rabbit IgG, mouse IgG, and goat IgG (Cell Signaling Technol. Danvers, MA, USA); Streptavidin-conjugated FITC and streptavidin-conjugated horseradish peroxidase (Carlsbad, CA, USA).

### 4.3. Cell Cultures

Human breast cancer cell lines (MCF-7 and MDA-MB-231) were a gift from Dr. V. Morandi (UERJ, RJ, Brazil) and certified (STR) by DNA Diagnostic Laboratory (UERJ, RJ, Brazil). MCF-7 and MDA-MB-231 cells were routinely maintained in DMEM supplemented with 10% FBS, NaHCO_3_ (3.7 g/L), HEPES (5.2 g/L), penicillin (0.5 U/mL), and streptomycin (0.5 mg/mL) and incubated at 37 °C in a humidified atmosphere of 5% CO_2_. For experiments, MCF-7 and MDA-MB-231 cells were grown to confluence and then detached with trypsin (0.1%)/EDTA (0.01%). To generate conditioned medium, MDA-MB-231 cells were grown in the same medium described above to 90–100% confluence (4 × 10^6^ cells) in 75 cm^2^ culture flasks for 72 h.

### 4.4. Isolation of Immobilized Cell-Derived Matrices

Freshly deposited ECM produced by MCF-7 (MCF-ECM) or MDA-MB-231 (MDA-ECM) was obtained, as previously described [26]. Briefly, cells were seeded onto plastic 6-, 24-, or 96-well plates and grown for 72 h. Monolayers were disrupted with cold lysis extraction buffer (2.7 mM KCl, 1.4 mM KH_2_PO_4_, 68.3 mM NaCl, 8 mM Na_2_HPO_4_, 1 mM MgCl_2_, 1 mM CaCl_2_, 0.1% Triton X-100, and 100 mM NH_4_OH) for 3–5 min until the cells appearing floating in the medium. Cell debris was washed twice with cold PBS-Ca^2+^, and the wells were saturated with 0.1% BSA/PBS-Ca^2+^ for 1 h at 37 °C immediately before use. Analysis under the light microscope showed that after this procedure, only the cell-derived ECM remained on the bottom of the wells, as described earlier [39].

### 4.5. Stimulation of MCF-7 Cells to Epithelial-Mesenchymal Transition (EMT)

MCF-7 cell suspension in DMEM 5% FBS medium (2 × 10^5^ cells/well) was seeded onto freshly immobilized extracellular matrices derived from MCF-7 and MDA-MB-231 cells. MCF-7 cells seeded onto their own matrix and stimulated with TGF-β1 (10 ng/mL) were used, respectively, as control and positive controls for EMT.

### 4.6. Analysis of ECM Composition by Indirect ELISA

The composition of deposited matrices from different cell types was analyzed by indirect ELISA, as previously described [39]. Absorbance was measured at 490 nm in an automatic ELISA microplate reader (Thermo Scientific, Waltham, MA, USA).

### 4.7. Immunofluorescence of E-cadherin and Confocal Microscopy 

Immobilized MCF-ECM and MDA-ECM were obtained on sterile glass coverslips. MCF-7 cells (7 × 10^4^ cells/well) were then cultured onto these matrices for 72 h. For the inhibition of integrin activation, MCF-7 cells (7 × 10^4^ cells/well) were treated with 1 μM RGD peptide (Arg-Gly-Asp) for 72 h. After 72 h, cells were fixed with 4% paraformaldehyde in sucrose/PBS solution for 20 min at room temperature, permeabilized with Triton X-100 (0.1%)/PBS for 5 min, and blocked with 5% BSA for 30 min. For the detection of E-cadherin by immunofluorescence, the slides were incubated with rabbit monoclonal anti-E-cadherin (1:400) overnight at 4 °C, washed and then incubated with the appropriate secondary antibody for 1 h, followed by incubation with Alexa Fluor 555 at 4 °C for 1 h. Finally, slides were mounted using ProLong Gold antifade reagent with 4,6-diamidino-2-phenylindole (DAPI) for nuclear staining. Coverslips were examined under an Olympus BX40 microscope equipped for epifluorescence at 60× magnification. Fluorescence intensity was measured at intercellular junctions on the surface of MCF-7 cells (white asterisks). The result showed the mean ± SD of the relative fluorescence intensity, measured on Icy bioimage analysis version 1.9.5.1 and Excel. 

For confocal images, MCF-7 cells were labeled with TRITC-phalloidin (1:400) overnight at 4 °C. Then, the sections were blocked with 5% BSA for 30 min and incubated with mouse monoclonal anti-vinculin (1:200) overnight at 4 °C. Coverslips were mounted and examined under an Olympus FV1000 Inverted Confocal IX81 at 60× magnification. Vinculin colocalized with actin (yellow points-white asterisks) was quantified by counting five random fields at the 60× magnification, for each treatment condition. The result showed the mean ± SD of the number colocalized actin/vinculin per cell in percentage. Vinculin and actin colocalization were measured on Icy bioimage analysis version 1.9.5.1 and Excel.

### 4.8. Migration Assay

MCF-7 (2 × 10^5^ cells/well) cells incubated on different cell-derived matrices for 72 h were harvested and counted in a hemocytometer after staining with trypan blue. A total of 7 × 10^4^ cells/well were added onto the upper membrane of each insert of Transwell^®^ permeable supports (8-μm pore size filter membranes; Becton Dickinson) assembled in a 24-well plate (Falcon) and allowed to migrate for 72 h at 37 °C and 5% CO_2_. Then, non-migrating MCF-7 cells were removed with cotton swabs, while migrating cells on the lower surface of filters were fixed and stained with a panoptic kit for counting. Migrating cells were quantified as the number of cells that migrated through the insert, counted in 10 fields in an inverted microscope (Olympus IX71) at 40× magnification.

### 4.9. Nuclear Extract

MCF-7 cells (2 × 10^5^ cells/well) were seeded for 72 h on MDA-ECM or their own matrices. Then, cells were lysed in ice-cold buffer A (10 mM HEPES, pH 7.9, 10 mM KCl, 0.1 M EDTA, 0.1 M, EGTA, 1 mM DTT, and 0.5 mM PMSF), and then Nonidet P-40 was added to a final concentration of 0.5% (*v*/*v*). Nuclei were collected by centrifugation (1810× *g* for 5 min at 4 °C). The nuclear pellet was suspended in ice-cold buffer C (20 mM HEPES, pH 7.9, 400 mM NaCl, 1 mM EDTA, 1 mM EGTA, 1 mM DTT, 1 mM PMSF, 1 µg/mL pepstatin, 1 µg/mL leupeptin, and 20% (*v*/*v*) glycerol) and incubated for 30 min. Nuclear proteins were collected in the supernatant after centrifugation (12,000× *g* for 10 min at 4 °C); then, the nuclear extracts were denatured in sample buffer (50 mM Tris-HCl, pH 6.8, 1% SDS, 5% 2-ME, 10% glycerol, and 0.001% bromophenol blue) and assayed in SDS-PAGE.

### 4.10. SDS-PAGE and Western Blot

MCF-7 cells (2 × 10^5^) were seeded for 72 h on MDA-ECM or MCF-7-ECM. To block TGF-βRI signaling, cells were incubated for 72 h with 15 µg/mL SB431542, a potent and selective inhibitor of TGF-β superfamily type 1 activin receptor-like kinase (ALK) receptors. To inhibit integrin activation, MCF-7 cells (2 × 10^5^ cells/well) were seeded on MCF-ECM and MDA-ECM, and after cell adhesion, RGD peptide (1 μM) or Kistrin disintegrin (0.4 μM) was added to cultures, which were then incubated in the presence or absence of TGF-β1 for 72 h. Then, MCF-7 cells were suspended in lysis buffer (50 mM Tris-HCl, pH 7.4, 150 mM NaCl, 1.5 mM MgCl_2_, 1.5 mM EDTA, Triton X-100 (1%, *v*/*v*), glycerol (10%, *v*/*v*), and the following protease inhibitors: 1 mM phenylmethylsulfonyl fluoride (PMSF), 1 mM benzamidine, and 1 μM soybean trypsin inhibitor). Total protein concentration was determined by BCA, according to the manufacturer’s protocol. Cell lysates were denatured in sample buffer (50 mM Tris-HCl, pH 6.8, 1% SDS, 5% 2-mercaptoethanol, 10% glycerol, 0.001% bromophenol blue) and boiled for 5 min. Samples (20 μg total protein) were resolved on reducing 10% SDS-PAGE, and proteins were transferred to polyvinylidene difluoride (PVDF) membranes for western blot analysis. Rainbow^TM^ molecular weight markers were run in parallel to estimate molecular weights. Membranes were blocked with 5% BSA and Tween-20 0.5%. Primary antibodies were used for immunoblotting. After extensive washing in Tween-TBS, membranes were incubated for 2 h with the appropriate secondary biotin-conjugated antibody (1:5000). Immunoreactive proteins were visualized using the ECL system. Band densitometry was quantified using Photoshop software (Adobe Systems, San Jose, CA, USA), and values were expressed as arbitrary units (AU).

### 4.11. qRT-PCR Analysis

MCF-7 cells (2 × 10^5^ cells/well) were incubated for 48 h onto MDA-ECM or on their own ECM. Total RNA was extracted using an RNeasy mini kit. cDNA was generated from 1 μg RNA using a high capacity cDNA reverse transcription kit. cDNA was amplified with human gene-specific primers for *TWIST* and β-actin. Each candidate gene was internally normalized against β-actin. The relative quantitative value was expressed by the 2^−∆∆*C*t^ method. qPCR was performed using a 7500/7500 fast real-time PCR system (Applied Biosystems, Foster City, CA, USA), and amplicons were quantified using an SYBR^®^ Green PCR master mix kit (Applied Biosystems, Foster City, CA, USA). PCR was performed with a program of 5 min at 95 °C and then 45 cycles at 94 °C (30 s), 60 °C (30 s) and 72 °C (30 s), followed by a standard curve of denaturation.

### 4.12. Statistical Analysis

All the plots inserted in figures represent the mean of at least three independent experiments carried out on different days to reduce any bias. Statistical analysis, unless otherwise stated, was assessed by ANOVA and Student’s *t*-test, with *p* < 0.05 taken as statistically significant.

## 5. Conclusions

Our data showed that the matrix produced by highly metastatic human breast cancer cells (MDA-MB-231) activated intracellular signaling pathways related to αvβ3 integrin and potentiated canonical TGF-β receptor signaling, inducing an EMT-like phenotype and migration in MCF-7 cells, likely favoring breast cancer metastasis. We believe that this data might contribute to unravel what happens in breast cancer relapses, even in patients that were classified as having luminal type tumors, which are known in the literature to have a better outcome, although this does not happen in all patients. These findings provided novel potential target molecules for antitumor and antimetastatic therapies and also represented an important contribution to the understanding of the adhesion-related mechanisms involved in EMT.

## Supplementary Materials

Supplementary materials can be found at https://www.mdpi.com/1422-0067/21/8/2995/s1.

## Abbreviations

MCF-ECM	Extracellular matrix-derived from the MCF-7 cell line
MDA-ECM	Extracellular matrix-derived from the MDA-MB-231 cell line
