# Supplementary material for: Extracellular Matrix Derived from High Metastatic Human Breast Cancer Triggers Epithelial-Mesenchymal Transition in Epithelial Breast Cancer Cells through αvβ3 Integrin
Source: Int J Mol Sci. 2020 Apr 23;21(8):2995. doi: 10.3390/ijms21082995 (PMC7216035; doi:10.3390/ijms21082995)
Supplement: Supplementary file 1 [file ijms-21-02995-s001.pdf]

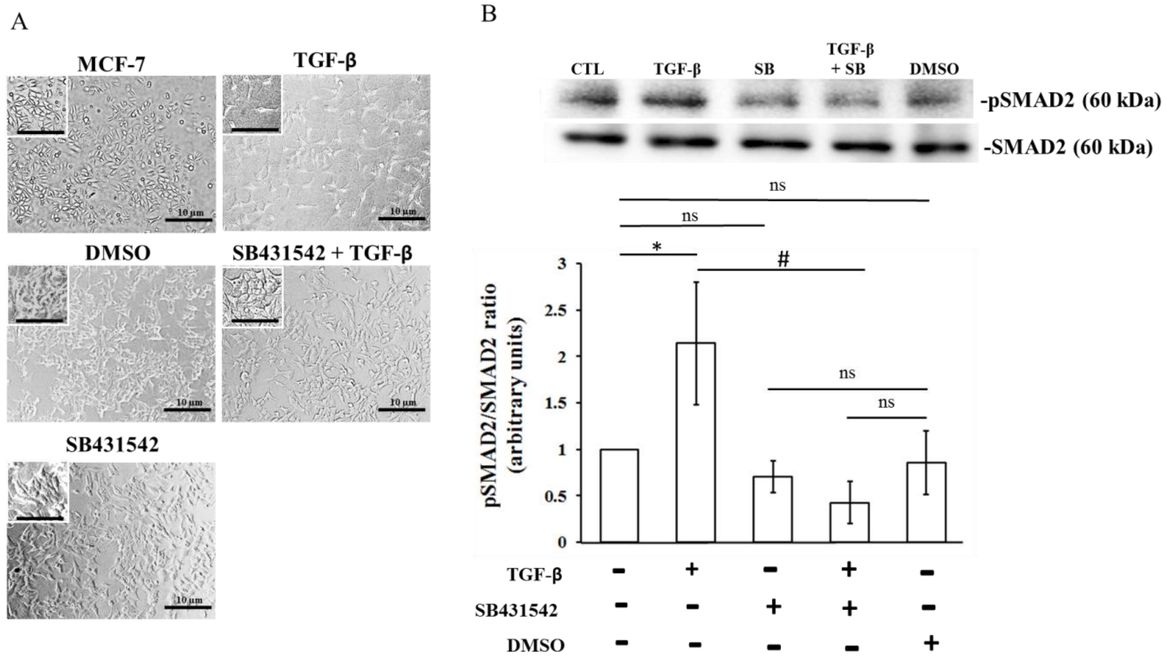

Figure S1. Inhibition of TGF-β receptor decreased TGF-β canonical signaling pathway and recovered epithelial morphology. (**A**) MCF-7 cells were cultured in standard conditions for 72 h with or without TGF-β1 and in the presence or absence of the TGF-β receptor inhibitor SB431542 (15 µg/mL). Cell morphology was analyzed and representative images were obtained at 40x magnification. Scale bar: 10 µm. (**B**) MCF-7 cells lysates were immunoblotted with anti-p-SMAD2 and anti-SMAD2 antibodies. The results are shown as the mean fold increase relative to the control (*$p < 0.05$); MCF-7 cells treated with TGF-β (#$p < 0.05$) calculated from 3 individual experiments.

A

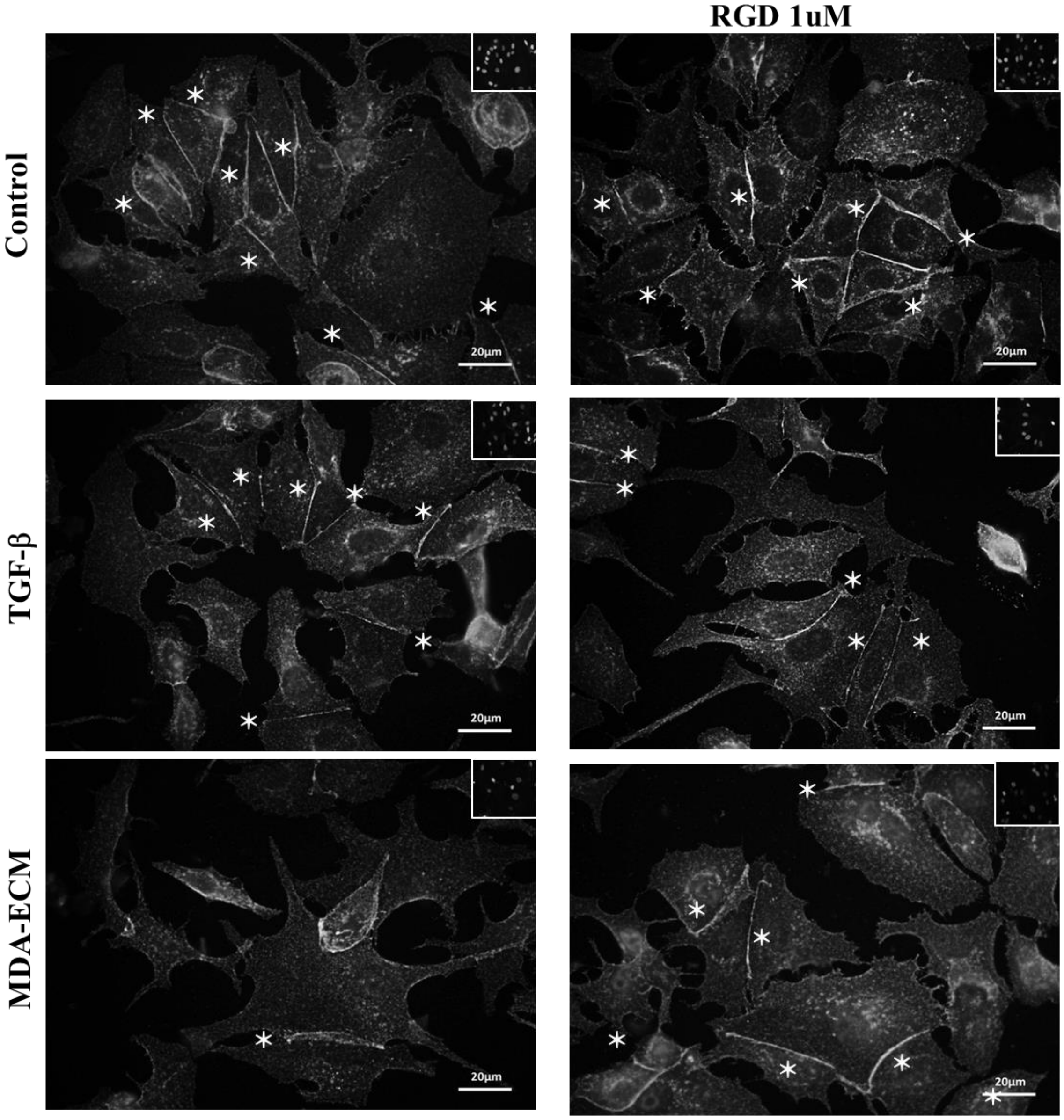
RGD 1uM
Control
TGF-β
MDA-ECM
20μm

B

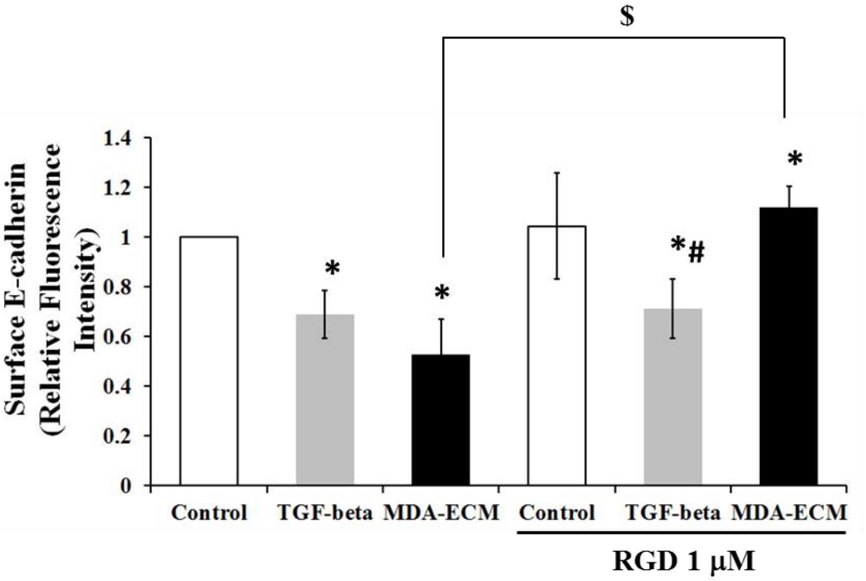
$
Surface E-cadherin
(Relative Fluorescence
Intensity)
1.4
1.2
1
0.8
0.6
0.4
0.2
0
*
*
*#
*
Control
TGF-beta
MDA-ECM
Control
TGF-beta
MDA-ECM
RGD 1 μM

Figure S2. Integrin signaling modulates E-cadherin–mediated adhesion of MCF-7 cells cultured on MDA-ECM in presence of RGD peptide. MCF-ECM and MDA-ECM were obtained as described in the Methods section. MCF-7 cells were cultured on their own ECM with or without TGF-β1 or on MDA-ECM and treated with RGD for 72 h (1 μM). (**A**) Effect of RGD treatment on E-cadherin expression and localization in MCF-7 cells cultured on MDA-ECM or MCF-ECM with or without TGF-β1. Representative images were captured on fluorescence microscopy at 60x magnification. (Asterisks: E-cadherin-mediated cell-cell contacts). Scale bar: 20 μm. (**B**) Representative graph showing the relative fluorescence intensity of E-cadherin in intercellular junctions. The results are shown as the mean fold increase relative to controls: MCF-ECM (*$p < 0.05$); MCF-ECM + RGD (#$p < 0.05$) and MDA-ECM ($\$p < 0.05$).
